# Papular lesion occurring within a longstanding warty plaque, in skin of colour Fitzpatrick type 4–5

**DOI:** 10.1002/ski2.328

**Published:** 2024-01-08

**Authors:** Joelle Teoh, Alex Gan, Joshua Ramalingam, Somaia Elsheikh, Richard Jerrom

**Affiliations:** ^1^ Nottingham University Hospitals NHS Trust Nottingham UK

## Abstract

A 23‐year‐old man of South Asian descent, Fitzpatrick type 4–5 skin, usually fit and well, presented with a 6‐month history of a darkly‐pigmented papular lesion growing within a pre‐existing warty plaque on the left parietal scalp, present since birth. The base plaque measured 30 × 10 mm, whilst the new papule measured approximately 3 × 3 mm. These were asymptomatic and there was no preceding trauma to the area. Examination revealed a pearlescent darkly pigmented papule, growing within a warty pink‐yellow hairless plaque. Dermoscopy showed non‐specific features with evidence of some disorganized vasculature. A punch excisional biopsy of the papular lesion was obtained, histopathology indicated a polypoid lesion with basaloid nests in a superficial and nodular distribution extending to the superficial dermis. The base plaque was then completely excised, showing dermal scarring related to the previous excision, along with the presence of large sebaceous glands, heterotopic apocrine glands, defective hair follicles, acanthosis and epithelial papillomatosis. This is a case of a basal cell carcinoma (BCC) arising within a sebaceous naevus on the scalp, in a skin of colour patient. Sebaceous naevi (SN), also known as naevus sebaceous of Jadassohn, are benign hamartomatous malformations comprised of predominantly sebaceous glands. SN appear most commonly on the scalp, and start off as smooth yellowish well‐circumscribed plaques in infancy which then develops a verrucous appearance in adolescence due to hormonally‐driven maturation of sebaceous and apocrine glands. It is well known that benign neoplasms of various lineages of differentiation including follicular, sebaceous, apocrine or eccrine, may arise within SN. Malignant neoplasms occurring within SN almost exclusively occur in adults, and arise in about 2.5% of lesions. BCCs are the most common among these, and occur in 0.8% of SN. Other malignant tumours such as squamous cell carcinoma, sebaceous carcinoma, microcystic adnexal carcinoma and porocarcinoma can also arise within SN, but these are rarer. Our case is notable as our patient had Fitzpatrick skin type 4–5, hence may have been perceived to have a lower risk of developing BCCs. We hope that this report will highlight that BCCs do arise even in skin of colour.

## Graphical abstract

Sebaceous naevi (SN), also known as naevus sebaceous of Jadassohn, are benign hamartomatous malformations comprised of predominantly sebaceous glands. Malignant neoplasms occurring within SN almost exclusively occur in adults, and arise in about 2.5% of lesions. Our case is notable as our patient had Fitzpatrick skin type 4–5, hence may have been perceived to have a lower risk of developing basal cell carcinomas (BCCs). We hope that this report will highlight that BCCs do arise even in skin of colour.

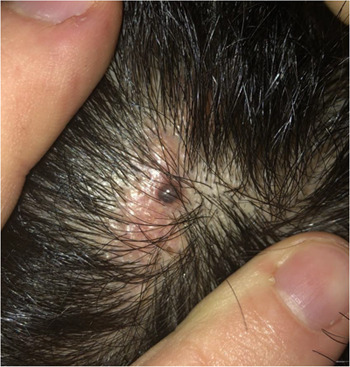

## 1 INTRODUCTION

A 23‐year‐old man of South Asian descent, Fitzpatrick type 4–5 skin, presented with a 6‐month history of a darkly‐pigmented papular lesion growing within a pre‐existing warty plaque on the left parietal scalp, present since birth. The base plaque measured 30 × 10 mm, whilst the new papule measured approximately 3 × 3 mm. These were asymptomatic and there was no preceding trauma to the area. He was a medical student, usually fit and well, with an unremarkable family history.

**FIGURE 1:**
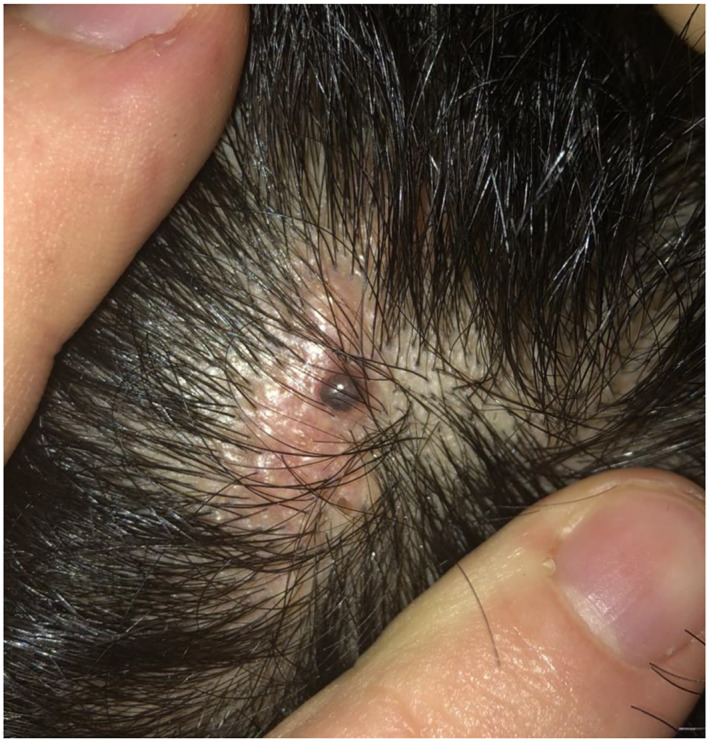
Papular pearlescent darkly pigmented lesion, growing within a warty pink‐yellow hairless plaque over the left parietal scalp.

Examination revealed a pearlescent darkly pigmented papule, growing within a warty pink‐yellow hairless plaque (Figure 1). Dermoscopy showed evidence of some disorganized vasculature, but otherwise non‐specific features.

## 2 HISTOPATHOLOGICAL FINDINGS

**FIGURE 2:**
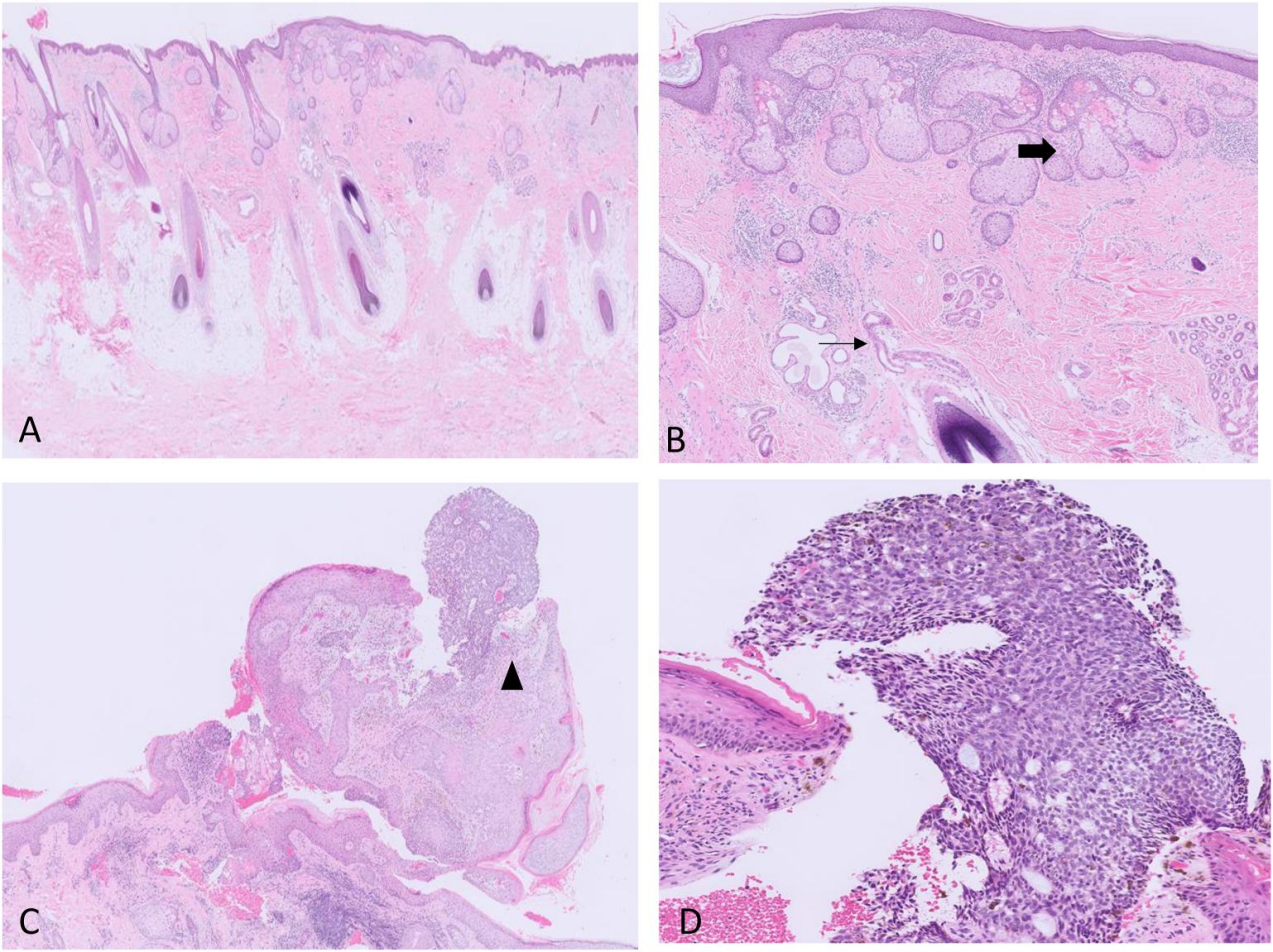
(a and b) Represent the plaque lesion. Histology shows hair‐bearing skin with sebaceous hyperplasia (thick arrow) and eccrine/apocrine changes (thin arrow). (c and d) Represent the new papular lesion. Histology shows nodular basal cell carcinoma (arrow head) arising in a background of papillomatous epidermal hyperplasia. Haematoxylin and eosin stain (a and c) 10× magnification and (c and d) 20× magnification.

A punch excisional biopsy of the new papular lesion was taken from the plaque. Histopathology indicated a polypoid lesion with basaloid nests in a superficial and nodular distribution extending to the superficial dermis (Figure 2). Subsequently, the base plaque was completely excised showing dermal scarring related to the previous excision, along with the presence of large sebaceous glands, heterotopic apocrine glands, defective hair follicles, acanthosis and epithelial papillomatosis.

## 3 DIAGNOSIS

Basal cell carcinoma arising within a sebaceous naevus on the scalp, in a skin of colour patient.

## 4 DISCUSSION

Sebaceous naevi (SN), also known as naevus sebaceous of Jadassohn, are benign hamartomatous malformations, comprised of predominantly sebaceous glands. These typically present from birth or early childhood and grow proportionally as the child grows. About 0.3% of all newborns may have SN, with an equal incidence in males and females. This is due to post‐zygotic somatic mutations involving the Ras protein family.^1^ SN appear most commonly on the scalp, but can happen anywhere else on the body, and start off as smooth yellowish well‐circumscribed plaques in infancy and then develops a verrucous appearance in adolescence due to hormonally‐driven maturation of sebaceous and apocrine glands. They persist into adulthood if not treated or excised.^2^ Occasionally, they can appear as part of syndromic disorders, such as Schimmelpenning Feuerstein Mims syndrome and phakomatosis pigmentokeratotica.^3^

It is well known that neoplasms of various lineages of differentiation including follicular, sebaceous, apocrine or eccrine, may arise within SN, and these are usually benign. Most common among these are trichoblastoma, syringocystadenoma papilliferum, and trichilemmoma.^3^

Malignant neoplasms occurring within SN almost exclusively occur in adults, and arise in about 2.5% of lesions. Basal cell carcinomas are the most common among these, and occur in 0.8% of SN. Our case is notable as our patient had Fitzpatrick skin type 4–5, hence may have been perceived to have a lower risk of developing BCCs. We hope that this report will highlight that BCCs do arise even in skin of colour. Other malignant tumours such as squamous cell carcinoma, sebaceous carcinoma, microcystic adnexal carcinoma and porocarcinoma can also arise within SN, but these are rarer.^4^, ^5^, ^6^

Due to the risk of malignant transformation, some specialists recommend complete surgical excision, but there is currently no clear evidence to support an optimal timing for this. As SN expand during adolescence, some argue that surgical removal before this phase is desirable. Other treatment measures such as laser or photodynamic therapy may result in incomplete clearance, hence risking recurrence. Some lesions may not be amenable to excision due to size and location, in which case watchful waiting would be preferred. Morbidity of surgical procedure in children is another factor to consider when determining the timing of excision.^2^, ^4^

## CONFLICT OF INTEREST STATEMENT

The authors declare no conflicts of interest.

## AUTHOR CONTRIBUTIONS

**Joelle Teoh**: Writing – original draft, Writing – review & editing (equal). **Alex Gan**: Writing – review & editing (equal). **Joshua Ramalingam**: Visualization (equal). **Somaia Elsheikh**: Resources (equal). **Richard Jerrom**: Writing – review & editing (equal).

## ETHICS STATEMENT

Not applicable.

## Data Availability

Data sharing not applicable to this article as no datasets were generated or analysed during the current study.
